# Quality assessment and quality control of deep soil mixing columns based on a cement-content controlled method

**DOI:** 10.1038/s41598-023-31931-y

**Published:** 2023-03-24

**Authors:** Jinyu Zuo, Baotian Wang, Wenwei Li, Shaoyang Han, Jiahui Wang, Fuhai Zhang

**Affiliations:** 1 Key Laboratory of Ministry of Education for Geomechanics and Embankment Engineering, Hohai University, Nanjing, People’s Republic of China; 2 Engineering Research Center of Dredging Technology, Hohai University, Changzhou, People’s Republic of China; 3 Department of Civil Engineering, Jiangsu Open University, Nanjing, People’s Republic of China

**Keywords:** Civil engineering, Chemical engineering

## Abstract

This paper presents a cement-content controlled method for quality assessment and quality control of the deep soil mixing (DSM) columns in slope reinforcement. The ethylene diamine tetraacetic acid (EDTA) titration method was modified and used for the cement content measurement of core samples, and the effects of curing conditions and curing period on the titration results were investigated. 35 DSM columns with different construction parameters were installed in the test section, and cement content and unconfined compression tests of field core samples were conducted. The relationship between the unconfined compressive strength (UCS) and cement content of DSM columns was formulated, and the quality of DSM columns with different construction parameters was assessed. The test results suggested that the failure strength of the field cores was approximately 15–55% lower than that of laboratory samples with the same cement content. In single columns, the coefficient of variation (CV) of cement content had a negative correlation with the average failure strength and a positive correlation with the coefficient of variation of failure strength. Bidirectional mixing method, lower penetration and withdrawal velocity, more mixing blades and larger number of mixings could improve the uniformity of the DSM columns.

## Introduction

The deep soil mixing (DSM) method is a widely used technology for in-situ ground improvement. Benefiting from its high improvement effect, low construction costs and minimal adverse impact on surroundings, the DSM method has great application in large-scale slope reinforcement engineering^1–3^. Deep mixing method includes both mechanical mixing and hydraulic mixing^4^, which use blades and high-pressure fluids to erode the soil and mix it with binder, respectively. The quality control (QC) and quality assurance (QA) of DSM columns is a question of common interest for engineers and constructors^5^. Hydraulic mixing involves far greater jetting pressure and more disturbance to the surrounding soil than mechanical mixing, so the quality of DSM columns depends largely on jetting parameters, such as jetting pressure, flow rate of the fluid and rod withdrawal rate^6,7^. In contrast, mechanical mixing requires attention to mixing parameters such as blade number, withdrawal rate, and rotation speed.

Quality assessment is an important part of QC/QA procedures and runs through the entire QA process^8,9^. In slope reinforcement, the weak parts of DSM columns have adverse impacts on the reinforcement and even lead to the failure of structures^10–12^. Both the failure strength and uniformity of DSM columns are key points in their quality assessment^13–15^. Core boring is the most commonly used and universally acknowledged technique in practical operation at present. The strength of DSM columns can be obtained directly and accurately by unconfined compression tests on drilled core samples^16^. However, the uniformity of DSM columns is commonly judged on visual observation of the drilled core, which is subjective and dependent on experience. The quality of DSM columns cannot be guaranteed well due to the lack of quantitative assessment of uniformity^17,18^. There are other quality assessment techniques including penetration, geophysical, loading test and nondestructive methods^19^. Because of less experience and the lack of a direct correlation between the measured data and DSM uniformity, the application of these methods is limited in slope reinforcement^20^.

For DSM columns using cement as a binder, the cement content of core samples can be used as an important index to represent their quality. Numerous laboratory tests have shown that the strength of cement-stabilized soil is significantly correlated with its cement content^21,22^, water content^23^, curing time, and curing condition^24,25^. For example, a low cement content usually means low failure strength. On the other hand, the uniformity of mixing can be judged directly by the distribution of cement content in single DSM columns, where dispersed distribution means poor uniformity of mixing. Unlike strength growing, the amount and distribution of cement slurry is commonly constant in DSM columns, and therefore the cement content has the potential for the quality assessment immediately after mixing construction. However, due to differences in curing conditions and mixing processes, the strength and uniformity characteristics of DSM columns in field tests are very different from those in laboratory tests. It is necessary to assess the feasibility of the cement content index for the QA/QC in field through comparison with the strength index.

The ethylene diamine tetraacetic acid (EDTA) titration method was used for the cement content measurement of core samples. The effect of soil properties, cement types, sieving size, frequency and amplitude of oscillation on EDTA titration results has been well understood^26^. The curing period is another extremely important factor worth exploring. It was suggested that establishing multiple EDTA standard curves for each curing period to obtain accurate cement content. However, due to the uncertainty of the titration time in practice, the establishment of multiple EDTA standard curves implies a large amount of work.

A cement-content controlled method is presented and used for quality assessment and quality control of the DSM method in this study. A total of 35 DSM columns were installed by mechanical mixing using blade. The effects of curing conditions and curing period on EDTA titration results were investigated in the laboratory. The cement content and unconfined compressive strength (UCS) of core samples were measured to assess the quality of DSM columns with different construction parameters. The correlations between the strength and uniformity of DSM columns with cement content were analyzed.

## Sampling area and materials

**Figure 1:**
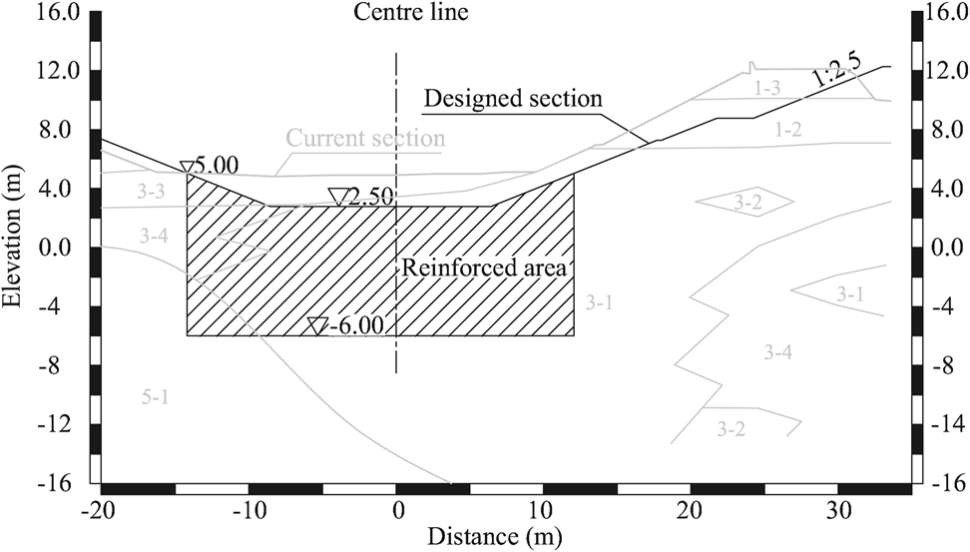
The representative cross-section of the test site on the Jiuxiang River.

A selected test section was constructed on the Jiuxiang River in Nanjing. The representative river cross-section of the test section is shown in Fig. 1. The engineering properties of the soils are shown in Table 1. The 3-1 silty clay loam is the key layer controlling the stability of the slope, which is characterized by high moisture content, low strength and large thickness. DSM columns were mainly installed in 3-1 soft silty clay for slope reinforcement. The designed UCS of the DSM columns was required to reach 0.8 MPa at 28 days.

**Table 1:** Engineering properties of soils at the test site.

No.	Soil layer name	γ (kN/m^3^)	*G*_*s*_	*e*	*w* (%)	*w*_*l*_ (%)	*w*_*p*_ (%)	α_1–2_ (Mpa^−1^)
1–2	Silty loam	19.4	2.72	0.75	25.1	31.1	18.6	0.38
3–1	Silty clay loam	18.2	2.73	1.08	37.9	34.9	21	0.61
3–3	Clay	16.0	2.75	1.86	66.8	45.4	25.4	1.90
3–4	Silty loam	19.1	2.72	0.87	30.9	33.0	19.8	0.39

*γ* unit weight,* G*_*s*_ specific gravity, *e* void ratio, *w* natural water content,* w*_*l*_ liquid limit,* w*_*p*_ plastic limit, *α*_*1–2*_ coefficient of compressibility.

Cement is widely used and has good improvement effect in DSM construction. 42.5 ordinary Portland cement was selected as a binder in the laboratory test, field test and production. Its properties are listed in Table 2.

**Table 2:** Chemical, physical and mechanical properties of 42.5 Portland cement.

Magnesium oxide (%)	Sulfur trioxide (%)	Loss of ignition (%)	Fineness (%)	Initial time of setting (min)	Final time of setting (min)
2.43	2.89	1.02	1.2	135	175

## Experimental procedure

The quality control and quality assurance (QC/QA) process commonly involves a laboratory test, field test, quality control during construction and quality verification/assurance after construction. Benefitting from the development of construction equipment, the quality of binder can be well maintained, and the construction parameters can be well controlled, monitored and recorded in production. Hence, this study mainly focuses on the control process prior to production, including the laboratory test and the field test.

### Laboratory mix test

To determine a suitable content of binder to ensure the design strength^27^, laboratory mix test was carried out on 3-1 silty clay loam prior to the field test. Cement-stabilized soil specimens with cement content of 14%, 16% and 18% and water cement ratios of 0.45, 0.50 and 0.55 were prepared. To simulate the in-site curing condition as much as possible, the specimens were placed into the untreated soil for maintenance, water was sprayed regularly to maintain the water content of the untreated soil, and the indoor temperature was kept at 20 °C. The specimens were removed for unconfined compression test at 7, 14, 28 and 90 days. The sample preparation and test process can be found in Chen and Standard for geotechnical testing method^21,28^.

Based on the laboratory mix test results, a 16% cement content and 0.55 water-cement ratio were selected. The laboratory mix test is a preliminary work of this study, and the details of the test results and the binder selection process can be found in Chen^21^.

### Modified EDTA titration method

The EDTA titration method measures cement content by detecting free calcium ions Ca^2+^ in stabilized soil^29^. Calcium hydroxide [Ca(OH)_2_] and gels containing calcium were generated during the cement hydration reaction. NH_4_Cl weak acid solution was used to dissolve calcium Ca^2+^ in hydration products, where the steady gels cannot be dissolved. After that, the NaOH solution was used to adjust the pH of the supernatant liquid, meanwhile triethanolamine was added to isolate the interfering ions Fe^3+^ and Mg^2+^. EDTA disodium salt was finally added to capture Ca^2+^ from calconcarboxylic acid (C_21_H_14_N_2_O_7_S), and the solution changed from pink to blue.

**Figure 2:**
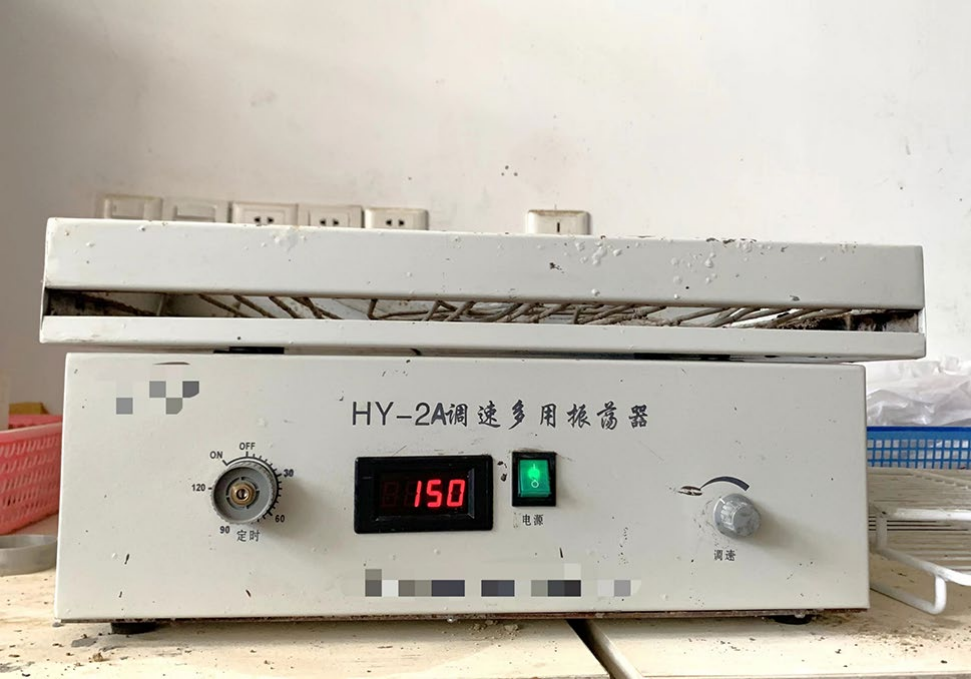
The HY-2A speed governing oscillator.

Based on previous research results, soil properties, cement types, sieving size, frequency and amplitude of oscillation affect EDTA consumption. In this study, 3-1 silty clay loam and 42.5 Portland cement were selected as the test soil and binder, respectively. The stabilized soil specimens were crushed and sieved through a 2-mm mesh. To keep the oscillation frequency and amplitude consistent, a reciprocating oscillator was used in the test, as shown in Fig. 2. The frequency and amplitude of oscillation were set as 150 times/min and 10 cm, respectively, and the oscillation time was 3 min. Although EDTA titration test requires some chemical agents and equipment, the cost of these agents used in a single test is not high, and the use of oscillator can also improve the test accuracy and reduces the labor cost. These additional test costs are generally acceptable.

Considering the influence of the water content, the EDTA consumption directly obtained by the titration test was converted to the standard EDTA consumption *V*_100_ (ml) of 100 g of dry stabilized soil using the following formula:

1 $$ V_{100} = \frac{100}{m}(1 + w)V, $$

where *m* (g), *w* (%) and *V* (ml) are the weight, water content and EDTA consumption of the stabilized soil specimens, respectively. The weight *m* of specimens in the test is 150 g.

To study the effect of the curing method, stabilized soil specimens with 15% and 20% standard cement content were prepared. As shown in Table 3, the water cement ratio of these specimens was 0.55. The specimens were treated with natural curing (the same curing conditions as in the laboratory mix test) and standard curing (the temperature was approximately 20 °C, and the humidity was approximately 90%). The standard EDTA consumption *V*_100_ was measured after 3, 7, 14, 28 and 60 days of curing.

**Table 3:** Details of specimens in laboratory test.

Curing method	Curing periods *T* (day)	Cement content *C*_*c*_ (%)	Water cement ratio
Standard curing	3, 7, 14, 28, 60	15%, 20%	0.55
Natural curing	1, 3, 5, 7, 14, 28, 60	0%, 10%, 15%, 16%, 18%, 20%, 25%, 30%	

To study the effect of the curing period on EDTA consumption, cement-stabilized soil specimens with eight kinds of cement content [0% (untreated soil), 10%, 15%, 16%, 18%, 20%, 25% and 30%] were prepared. The specimens were treated with natural curing, and the standard EDTA consumption *V*_100_ was measured after1, 3, 5, 7, 14, 28 and 60 days of curing.

### Field test

A DSM site was constructed on the selected test section. A total of 35 DSM columns (arranged in a 5 × 7 grid) were installed in the field site. The diameter of the DSM columns is 700 mm, and the length is approximately 10.5 m. The spacing between adjacent DSM columns is 1.1 m and 1.15 m (equivalent to a 30% replacement ratio). Based on the results of the laboratory mix test, DSM columns were produced using 42.5 grade Portland cement with 16% cement content (approximately 105 kg/m) and a 0.55 water-cement ratio. The mixing blade had a uniform rotation of 40 rpm. The quality of DSM columns is affected by various construction process parameters, including the mixing method, number of mixing blades, penetration and withdrawal speed and number of mixings. 35 DSM columns were divided equally into seven groups, and the design details of each group of columns are summarized in Table 4. For the columns of groups #G1 and #G3–#G7, the binder was injected during every penetration and withdrawal of the mixing machine except for the last withdrawal. For the #G6 columns with remixing at 6–9 m, the mixing shaft penetrated to 9 m again after the last withdrawal to 6 m, and then withdrew to the surface.

**Table 4:** Construction parameters of DSM columns.

Group	Mixing shaft speed (m/min)	Blade number	Number of mixings	Mixing method
G1	1.05, 1.05, 1.5, 1.5	10	Four-mixing-three-injection	Unidirectional
G2	0.6, 0.8	10	Two-mixing-two-injection	Bidirectional
G3	1.05, 1.5, 1.5, 1.5	8	Four-mixing-three-injection (G6 has a remixing at 6–9 m)	
G4	1.05, 1.5, 1.5, 1.5	10		
G5	1.05, 1.05, 1.5, 1.5			
G6	1.05, 1.05, 1.5, 1.5			
G7	1.05, 1.05, 1.8, 1.8, 1.8, 1.8		Six-mixing-five-injection	

Considering the difficulty of conducting strength tests on cement-stabilized soil at early ages, the tests were carried out on specimens after 28 days of curing. An XY-1 site investigation drill was used to drill DSM core samples. The drill location is approximately 1/3 of the column diameter away from the center of the DSM columns. To verify the correctness of the cement-content controlled method, unconfined compression test is carried out as well. Within the depth range of DSM columns, one specimen was selected per meter for the unconfined compression test in laboratory, and the crushed specimens were subjected to the EDTA titration test after further treatment. The crushed specimens were treated with natural curing if the EDTA titration test was not conducted in time. The unconfined compression test was carried at the curing time of 28 days, and the EDTA titration test was carried at the curing time of 28–31 days. Although the results of EDTA test are not at the same age, the cement content of DSM columns during curing is assumed to be usually constant.

## Results and discussion

### Modified EDTA titration method

#### Influence of curing method on EDTA consumptions

**Figure 3:**
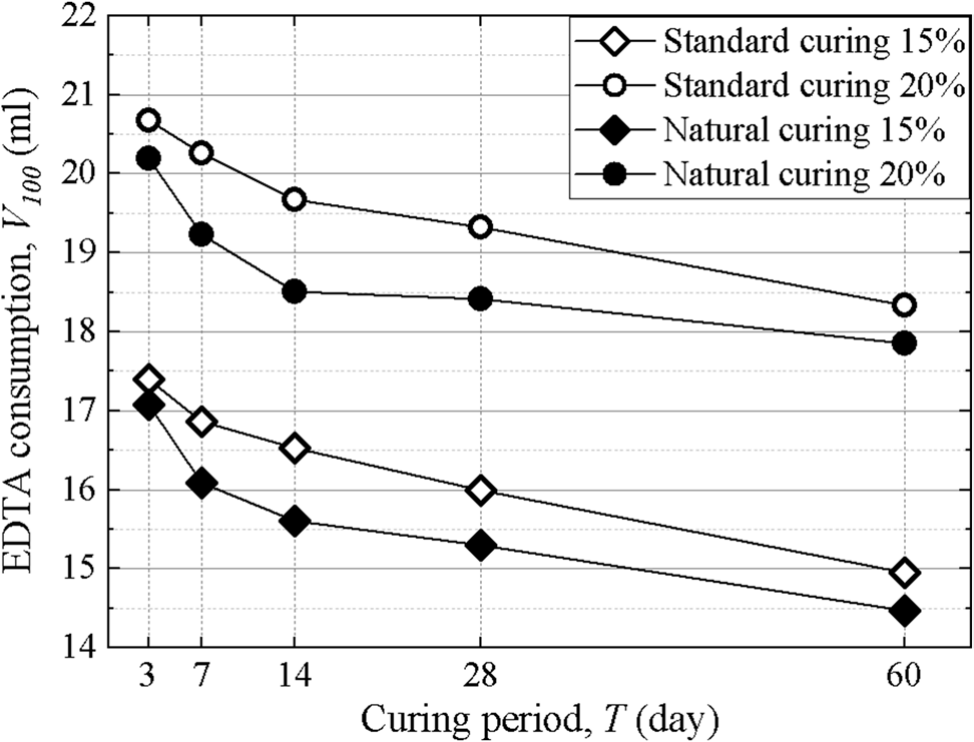
EDTA consumption of specimens under different curing methods.

Figure 3 shows the standard EDTA consumption *V*_100_ of stabilized soil specimens treated with different curing methods. For specimens with the same cement content, the EDTA consumption under standard curing conditions is significantly larger than that under natural curing conditions. This is because the surrounding soil in natural curing condition provides abundant ions and a suitable temperature and humidity for reactions, which results in a faster rate of cement hydration. In addition, with the growth of the curing period, the difference in EDTA consumption between the two curing methods increases first and then decreases. At the initial stage of the reaction, both curing methods can provide sufficient reaction conditions, so the difference is not large. With the progress of the reaction, the curing reaction of specimens with natural curing becomes more rapid, and the difference increases gradually. At the late reaction stage, the curing reaction of the two curing methods slows down and tends to be stable, and the difference decreases gradually.

**Figure 4:**
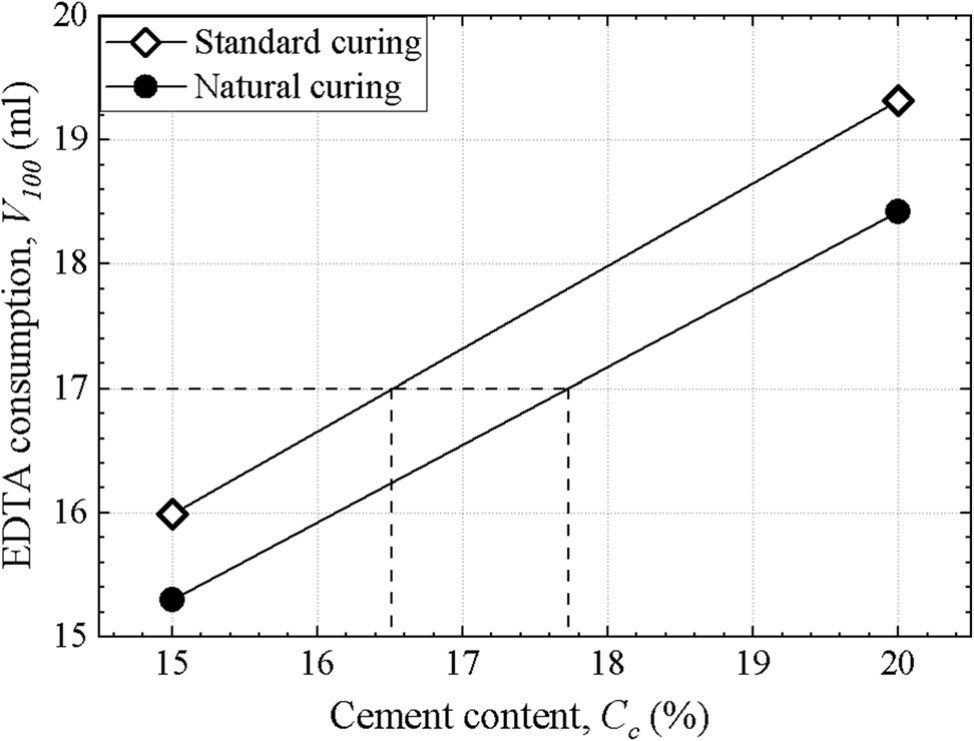
EDTA standard curves of different curing methods.

The EDTA standard curve is used to determine the cement content of stabilized soil. Figure 4 shows the EDTA standard curves of the two curing methods after 28 days of curing. There is a difference between the two curves resulting from the difference in EDTA consumption of the two curing methods. For example, a 28-day stabilized soil specimen has an EDTA consumption of 17.0 ml, and its cement content calculated by the curve of the natural curing is 17.7%, while it is 16.5% by the curve of standard curing. Natural curing can better simulate the substances and water exchange between stabilized soil and the surrounding environment and better conforms to the actual situation of DSM columns. The curve of standard curing underestimates the cement content of the specimen. Therefore, specimens with standard cement content should be treated with natural curing to obtain accurate EDTA standard curves.

#### Influence of curing period on EDTA consumptions

**Figure 5:**
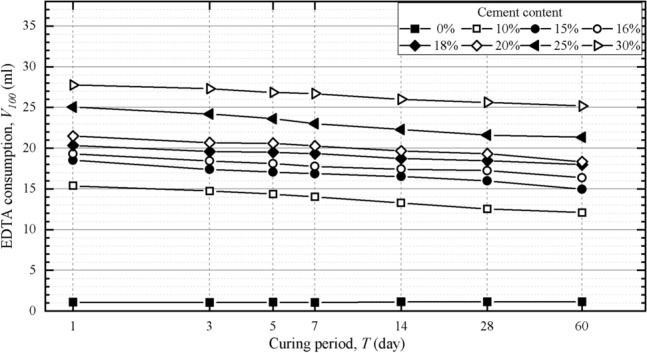
Variation of EDTA consumptions of different curing periods.

The EDTA consumptions of stabilized soil specimens with different cement content and curing periods are shown in Fig. 5. For specimens with the same curing periods, a higher cement content means that the specimen has more initial calcium, so the corresponding consumption of EDTA disodium salt is higher. For specimens with the same cement content, the EDTA consumption decreases with increasing curing period. EDTA consumption decreased rapidly in the early stage of the reaction, and the decreasing rate of EDTA consumption slowed down gradually. This is because as the cement hydration reaction proceeds, calcium ions existing in the form of stable gels become more abundant, and free calcium ions Ca^2+^ become less abundant. The ion concentration gradually decreases, and the reaction rate gradually slows down, which is consistent with the growth of the stabilized soil strength.

#### Calculation for cement content of specimens

**Figure 6:**
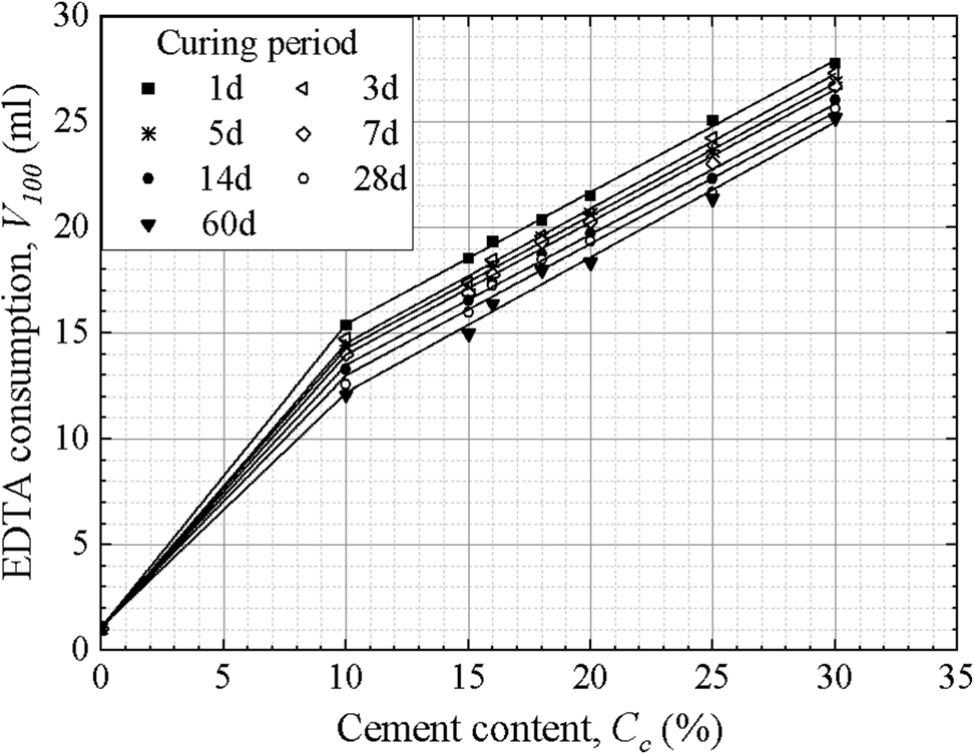
EDTA standard curves of the tested curing periods.

According to the test results, EDTA standard curves of the test curing periods (1, 3, 5, 7, 14, 28 and 60 days) can be obtained, as shown in Fig. 6. The dissolution capacity of ammonium chloride decreases with increasing cement content, so the standard curves were divided into two parts: a section of 0–10% and a section of 10% or higher cement content. Based on these standard curves, the cement content of specimens with the test curing periods can be calculated conveniently. However, core boring of DSM columns usually takes place after 28 days, and the operation time of the EDTA titration test may be postponed due to various factors in engineering. The cement content of specimens with other curing periods cannot always be calculated from these curves, meanwhile plotting EDTA standard curves of all possible curing periods increases the work cost.

The decrease characteristics of EDTA consumption shown in Fig. 5 indicates that EDTA consumption *V*_*100*_ (ml) has a logarithmic correlation with the curing period* T* (day), which can be expressed as

2 $$ V_{100} = {\text{k}}_{{1}} + {\text{k}}_{{2}} \ln (T + 1), $$

where k_1_ and k_2_ are coefficients related to the cement content. Based on Eq. (2) and any specified curing period, the EDTA consumptions of specimens with the 8 cement contents can be calculated, and then the required EDTA standard curve can be obtained.

Combining the linear relationship between EDTA consumption *V*_100_ (ml) and cement content *C*_*c*_ (%) shown in Fig. 6, EDTA consumption can be further expressed as

3 $$ V_{100} = {\text{k}}_{{1}} + {\text{k}}_{{2}} C_{c} + {\text{k}}_{{3}} \ln (T + 1) + k_{4} C_{c} \ln (T + 1), $$

where *T* (day) is the curing period, and k_1_, k_2_, k_3_ and k_4_ are constants depending on the soil type, binder type, curing conditions, and so on. Equation (3) in this study is

4 $$ V_{100} = \left\{ {\begin{array}{*{20}l} {{1}{\text{.10}} + {147}{\text{.9}}C_{c} - 8.94C_{c} \ln (T + 1)} \hfill & {0 \le C_{c} \le 10\% } \hfill \\ {{9}{\text{.63}} + 62.6C_{c} - 0.9\ln (T + 1) + 0.095C_{c} \ln (T + 1)} \hfill & {C_{c} > 10\% } \hfill \\ \end{array} } \right., $$

and the correlation coefficient R^2^ is 0.99.

By substituting the curing period *T* and the EDTA consumption *V*_100_ of the measured specimens into Eq. (4), the cement content of specimens with any curing period can be calculated efficiently. Based on the EDTA consumptions shown in Fig. 5, the actual cement contents of specimens are compared with the cement contents calculated from the Eq. (4). Their relative error of calculations is between − 5.27 and 6.26%, indicating that the fitting Eq. (4) is accurate. The strength of coring samples of DSM columns changes with the curing period, while the initial cement content after mixing is usually unchanged. Hence, the quality assessment based on cement content can be conducted immediately following the construction of DSM columns; by this time, the mixture of cement slurry and soil is still liquid and easy for boring. Furthermore, the quality assurance is brought forward, and unqualified columns can be handled instantly.

### Correlation between failure mechanism and cement content

**Figure 7:**
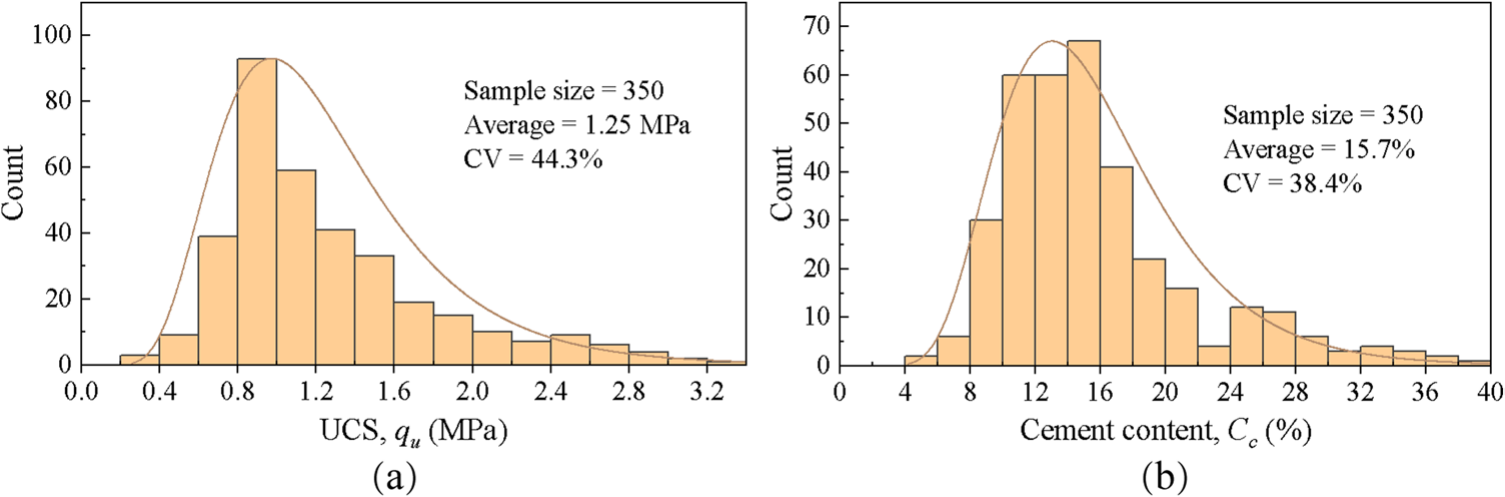
Histograms of UCS and cement content of core samples (**a**) UCS, (**b**) cement content.

Due to strict monitoring during construction, it can be considered that all DSM columns have nearly the same amount of cement, and the quality of columns is mainly controlled by the uniformity of mixing. A total of 350 core samples from 35 DSM columns were drilled in the field test section. The histograms of UCS and cement content of 350 core samples are shown in Fig. 7. The average and coefficient of variation (CV) of UCS and cement content of single columns are summarized in Table 5. It can be found that the UCS and cement content of the drilled core samples are characterized by lognormal distribution^20,30^. The UCS of the core samples is mainly concentrated between 0.6 MPa and 1.6 MPa, and the cement content is concentrated between 8 and 22%.

**Table 5:** Average and CV of UCS and cement content of single columns.

Group	Average of UCS (MPa)	CV of UCS (%)	Average of cement content (%)	CV of cement content (%)	Water content *w* (%)
G1	0.93–1.05	62.2–79.6	13.0–14.7	52.8–75.3	27.9–51.7
G2	1.08–1.19	48.3–67.6	14.5–16.0	45.7–56.2	26.7–43.1
G3	1.04–1.27	31.5–51.8	14.4–17.0	22.4–42.2	21.9–58.2
G4	1.11–1.35	31.7–43.8	15.1–16.6	24.3–47.4	21.7–49.3
G5	1.20–1.39	30.0–45.1	15.5–16.2	30.0–46.4	24.7–51.9
G6	1.20–1.44	24.5–39.7	15.6–17.1	27.6–43.5	21.6–51.5
G7	1.53–1.63	26.2–33.4	16.4–17.9	18.8–27.8	25.1–44.9

**Figure 8:**
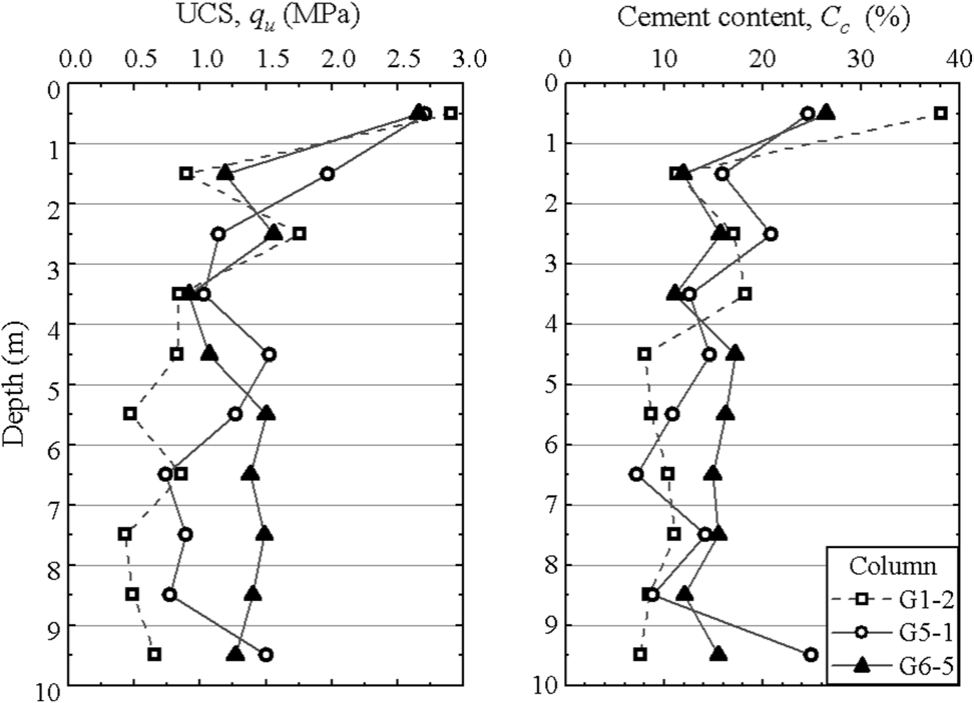
Distribution of the cement content and UCS with depth of typical columns.

**Figure 9:**
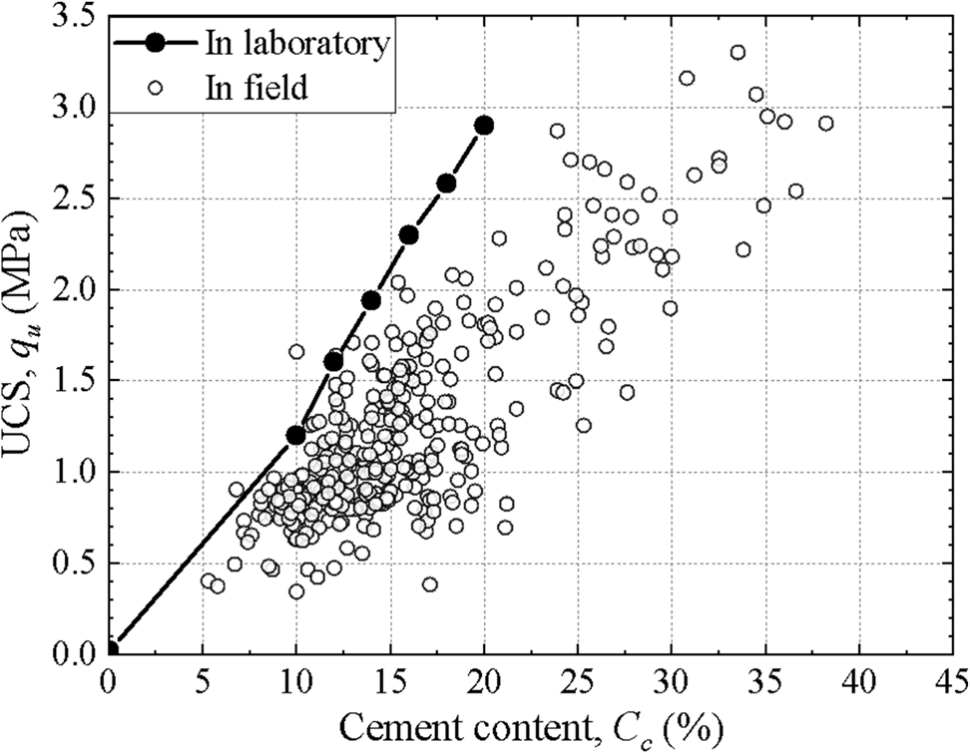
Relationship between UCS and cement content of specimens.

Figure 8 shows the distribution of the cement content and UCS of some typical columns. It can be found that the distribution of cement content with depth has a similar trend to that of UCS. The cement content and UCS of core samples are plotted in Fig. 9, including the results of field tests and laboratory tests. The higher the cement content of the sample, the higher the strength. The relationship between the strength and cement content indicate that the field strength is approximately 15% to 55% less than that of the corresponding laboratory samples. The difference in strength of laboratory and field specimens can be attributed to the differences in mixing methods, uniformity of mixing and curing conditions.

**Figure 10:**
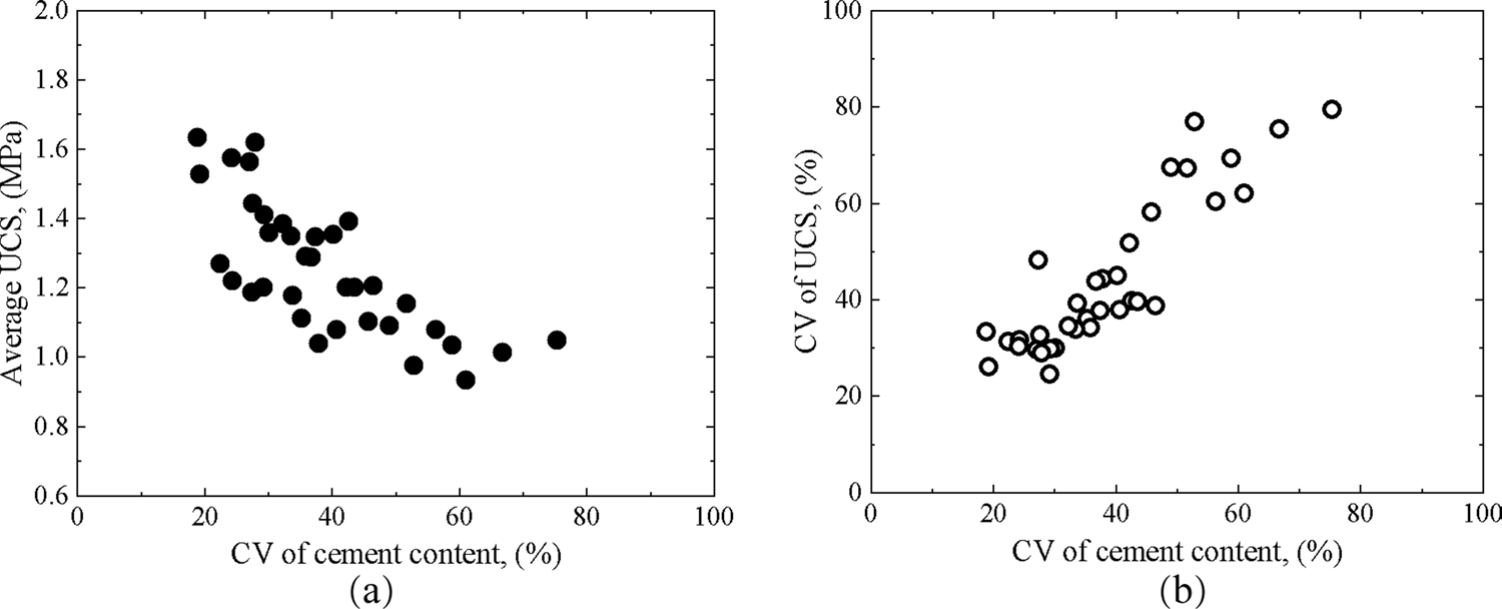
Relationship between UCS and CV of cement content of single columns (**a**) Average UCS, (**b**) CV of UCS.

The CV of cement content is an indicator of the uniformity of mixing. The higher the CV of the cement content in a single mixing column, the more discrete the distribution of the cement content, which means that the uniformity of mixing is worse. Figure 10a,b show the relationship between UCS and the CV of cement content of single columns. It can be found that the CV of cement content had a negative correlation with the average UCS and a positive correlation with the CV of UCS. When forming cement-stabilized soil, the injected cement slurry and soil are mixed together by a mixing blade, and sufficient cement slurry can react with the surrounding soil to form stable cement-soil masses with high strength. These stable masses alternate in space with the soil masses, which have less cement slurry and weak strength, forming the structure of the mixing column. The distribution of cement slurry is different in the mixing space due to different mixing uniformity. In a mixing column with poor uniformity of mixing, a large amount of cement slurry is concentrated in a few areas, while less cement slurry is distributed in most of the remaining areas. The coring samples with poor uniformity usually have low unconfined compressive strength even if these coring samples have sufficient cement content. Reflecting into the whole single column, the average strength of the samples is also low. The strength of drilled core samples also varies greatly, with a small part having higher strength and a large part having lower strength. The distribution of the strength of the overall DSM column is discrete, and the CV of the strength is higher.

### Influence of construction parameters on quality of DSM columns

#### Influence of mixing method on quality of DSM columns

**Figure 11:**
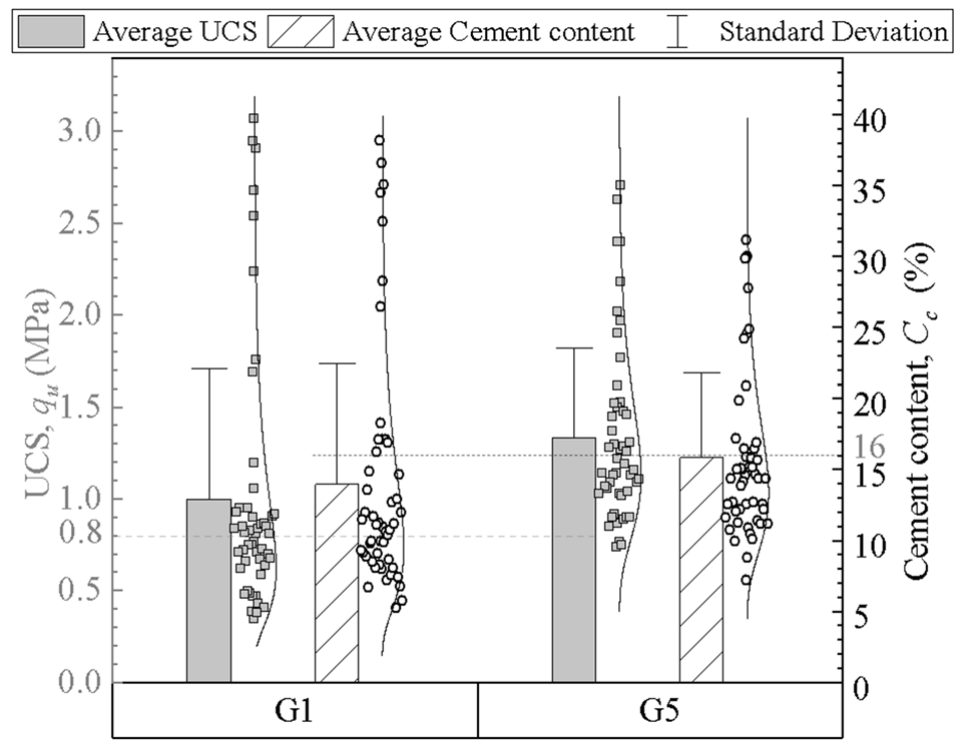
Cement content and UCS of the columns of groups #G1 and #G5.

The unidirectional mixing method and bidirectional mixing method were adopted for DSM columns of groups #G1 and #G5, respectively. The cement content and unconfined compression test results are listed in Table 5 and Fig. 11. The average cement content and average UCS of a single column with the unidirectional mixing method are 13.0–14.7% and 0.93–1.05 MPa, respectively, and those of the bidirectional mixing method are 15.5–16.2% and 1.20–1.39 MPa, respectively. Both the maximum cement content and the maximum UCS of unidirectional mixing method are greater than that of bidirectional mixing method, but the average value of unidirectional method is less. As shown in Fig. 8, the upper part of the unidirectional columns has high values of cement content and UCS, while the lower part has low values. Due to the joint action of earth pressure, pore water pressure and injecting pressure, the lower part of the cement slurry is squeezed to move up along the mixing shaft. The upper part of the unidirectional column has more cement slurry and higher strength, while the cement content and strength in the lower part are obviously lower than the design value. The CV of the cement content and strength show that the unidirectional mixing method has a weak mixing capacity and shallow reinforcement range. In the bidirectional mixing method, the internal and external mixing shaft drive two groups of blades to rotate in different directions, which can prevent the lower cement slurry from rising and mix cement slurry and soil more effectively^31^. Hence, the bidirectional mixing method is generally preferred in construction due to its good mixing quality and uniformity.

#### Influence of mixing shaft speed and blade number on quality of DSM columns

**Figure 12:**
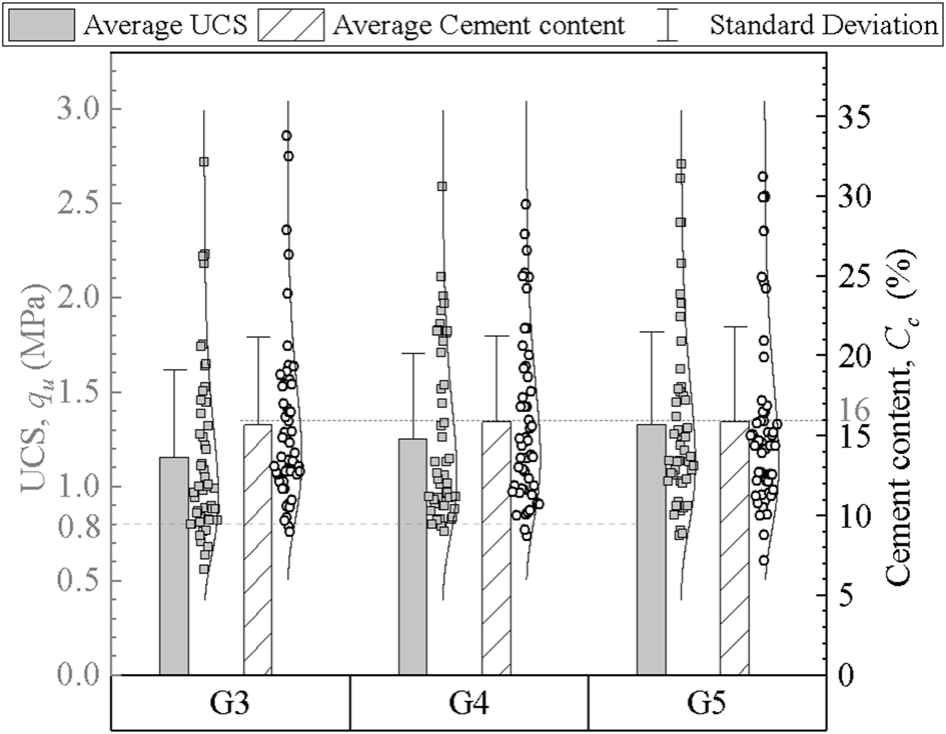
Cement content and UCS of the columns of groups #G3, #G4 and #G5.

Three sets of different mixing shaft speeds and blade numbers were adopted for columns of groups #G3, #G4 and #G5, as shown in Table 4. The cement content and strength results of the mixing columns are summarized in Fig. 12. It can be found that all three groups of columns have similar average cement content, but columns of groups #G3 and #G4 have lower average UCS and more dispersed distribution of cement content than #G5 columns. This is because these columns have fewer blades or faster penetration and withdrawal speeds, which results in the total mixing number of blades during 1 m of shaft movement being fewer, and the corresponding mixture of cement and soil being poor. The blade rotation number was an index with respect to the mixing shaft speed and blade number to evaluate the mixing degree. #G5 columns have larger blade rotation number than the other two groups, so their mixing degrees and test results are better as well. The comparison results show that both increasing the blade number and reducing the mixing shaft speed can improve the quality of DSM columns. Considering the efficiency and time cost of engineering construction, adding moderate mixing blades is the more recommended method.

#### Influence of number of mixings on quality of DSM columns

**Figure 13:**
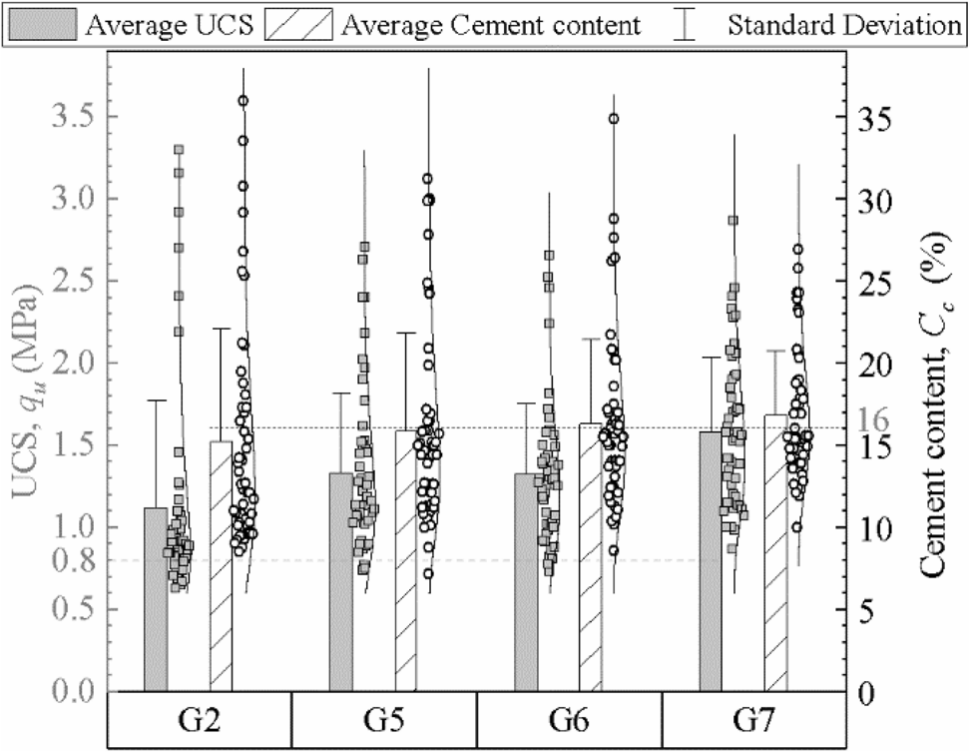
Cement content and UCS of the columns of groups #G2, #G5, #G6 and #G7.

Columns of groups #G2, #G5, #G6 and #G7 had different numbers of mixings, as listed in Table 4, and their construction times for a 10.5-m-long column were about 31 min, 34 min, 38 min and 44 min, respectively. Figure 13 presents the cement content and strength results of the four groups of columns. The strength and uniformity of columns with four-mixing-three-injection are better than those with two-mixing-one-injection, and the columns with six-mixing-five-injection have the lowest CV of cement content of 23.1% and the highest average strength of 1.58 MPa. The factors that contribute to these differences include the construction time and blade rotation number. Both the columns with four-mixing-three-injection and six-mixing-five-injection exhibit good quality, but the latter has more wear and tear on the mixing blades of and is more likely to cause damage to the mixing machine. As shown in Fig. 8, the 6–9 m part of #G5 columns has low UCS and cement content due to the changes of earth pressure and soil conditions. Compared with the #G5 columns, the #G6 columns have increased the mixing times of the weak part (6–9 m), and the uniformity of mixing had been improved. Adding local remixing at weak part of columns is an alternative to improve the mixing uniformity and quality of the weak part.

## Summary and conclusions

A cement-content controlled method was employed to assess and control the quality of DSM columns in this research study. The following conclusions can be drawn:

1. The modified EDTA titration method can be used for the convenient and accurate measurement of cement content. The curve obtained by standard curing underestimates the cement content of the column specimens. The laboratory test results indicated that EDTA consumption has a logarithmic correlation with the curing period and a positive correlation with cement content. The empirical equation developed based on these correlations can be used to calculate the cement content of cement-soil specimens for any curing period.
2. The failure strength of DSM columns in the field is positively correlated with the cement content. The strength of the field samples is approximately 15 to 55% lower than that of the laboratory samples with the same cement content. This can be attributed to the differences in mixing methods, uniformity of mixing and curing conditions.
3. The field test results revealed that the coefficient of variation of cement content is an indicator of the uniformity of mixing, having a negative correlation with the average strength and positive correlation with the CV of strength.
4. Many measures, including adopting the bidirectional mixing method, increasing the blade number, reducing the mixing shaft speed and adding the number of mixings, were demonstrated to improve the quality of DSM columns and prevent failures of reinforcement structures.

At this stage, only the influence of cement content on the quality of cement-stabilized soil is considered, and other factors such as water content and curing conditions can be considered in further study. Although quality assurance cannot be conducted due to the delay of large-scale construction, the potential of the cement-content controlled method has been demonstrated. The proposed method is an efficient and reliable alternative for the quality assessment and quality control of DSM columns in slope reinforcement.

## Data Availability

All data and models that support the findings of this study are available from the corresponding author upon reasonable request.
